# Comparative effectiveness of 10-week equipment-based pilates and diaphragmatic breathing exercise on heart rate variability and pulmonary function in young adult healthy women with normal BMI – a quasi-experimental study

**DOI:** 10.1186/s13102-023-00693-5

**Published:** 2023-07-11

**Authors:** Songül Adıgüzel, Dicle Aras, Mehmet Gülü, Monira I. Aldhahi, Abdulfattah S. Alqahtani, Sameer Badri AL-Mhanna

**Affiliations:** 1 Graduate School of Health Sciences, Ankara University, Ankara, Türkiye; 2 Department of Coaching Education, Faculty of Sport Sciences, Ankara University, Ankara, Türkiye; 3 Department of Sports Managemet, Faculty of Sport Sciences, Kırıkkale University, Kırıkkale, Türkiye; 4 Department of Rehabilitation Sciences, College of Health and Rehabilitation Sciences, Princess Nourah bint Abdulrahman University, P.O. Box 84428, Riyadh, 11671 Saudi Arabia; 5 Department of Rehabilitation Sciences, College of Applied Medical Sciences, King Saud University, 11433 Riyadh, Saudi Arabia; 6 Department of Physiology, School of Medical Sciences, Universiti Sains Malaysia, Kubang Kerian 16150, Kelantan, Malaysia

**Keywords:** Pilates, Breathing exercise, Heart rate variability, Respiration, Body composition

## Abstract

**Background:**

The positive effects of Pilates and slow-controlled breathing exercises on health are examined in different studies. The purpose of the study was to investigate the effects of 10-week equipment-based Pilates, slow-controlled breathing exercises, and a combination of both on heart rate variability (HRV), pulmonary function, and body composition (BC) in young adult healthy women with normal BMI.

**Methods:**

Forty female participants were assigned to either equipment-based Pilates group (PG), slow-controlled breathing exercise group (BG), equipment-based Pilates + breathing exercise group (PBG), and control groups (CG). Equipment-based Pilates exercise consists of training for two days a week and 50 min per day, and breathing exercises were done twice a week for 15 min a day for 8 weeks. In addition, PBG performed a 15-minute breathing exercise after each Pilates session. Pilates sessions were created with Reformer, Cadillac, Ladder Barrel, Chair Barrel, and Spine Corrector. On the other hand, breathing exercises were based on a controlled 5 s inhale and 5 s exhale cycles.

**Results:**

Before and after the implementation, pulmonary function, HRV, and BC parameters were measured. The body weight and BMI improved in PG and PBG, and the percent body fat decreased only in PBG (p < 0.05). Both PG and PBG noted significant changes in HRV indices SDSD, SDNN, TP, HF, and LF. However, the RMSSD was recorded higher in only PBG. Similar changes were found in pulmonary parameters. The FVC, FEV1, VC, IC, TV, MVV, and VE im-proved in PBG. PG showed increases in VC and TV. The only changes found in BG were PEF and ERV.

**Conclusions:**

The finding highlights the ample effect of combined breathing and Pilates exercise on HRV, pulmonary function and body composition which has important implications for health promotion.

## Background

Pulmonary functions, and body composition parameters have been considered as substantial indicators of health. The HRV defined as the fluctuation in the time intervals between successive heartbeats [1] which is accepted as a measure of neurocardiac function reflecting heart-brain interactions and autonomic nervous system (ANS) dynamics [2]. Good resting vagal-based HRV values are also accepted as indicators of the performance of executive functions such as attention and emotional processing controlled by the prefrontal cortex [1]. On the other hand, deterioration in HRV is considered a sign of the inability of the regulatory systems to adapt to changes and, therefore, disease or mortality [2]. Thus, HRV measurements are also used in the field of sport sciences to examine the acute and chronic responses to training. In a study in which a 12-week Pilates exercise program was performed, the improvements in SDNN, LF, and SD2 parameters of HRV were demonstrated among adult men [3].

Efficient pulmonary function is an important determinant of health. It has been reported that any regression in respiratory mechanisms may cause a decrease in exercise capacity and progressive deterioration in quality of life [4]. Thus, studies evaluated the effects of different type of exercises on pulmonary functions. For instance, swimmers were found to have greater respiratory values when compared with football players and sedentary subjects [5]. Another study claimed that performing Pilates in addition to the inspiratory training had better than performing only Pilates [6]. In a study stating that the combination of physical exercise and breathing exercise had a greater combined effect on pulmonary functions, forced expiratory volume showed greater improvement [7]. Pulmonary functions has also effects on HRV [8]. The heart speeds up and slows down depending on respiration via the vagus nerve [9]. The time-domain parameters of HRV increase during inspiration and decrease during expiration, and in general, the longest time-domain parameters of HRV and the largest amplitude in respiratory sinus arrhythmia are achieved at six respiratory frequencies per minute [10]. Therefore, slow-controlled breathing exercise has been used in scientific research to improve HRV. It has been reported that 3-month of slow-breathing exercise increased the vagal activity [11]. Similarly, the acute effects of slow breathing exercises resulted again in a higher level of vagal activity when compared with fast breathing [12].

Body composition is one of the health-related parameters of physical fitness and is associated with several diseases such as coronary artery disease, obesity, hypertension, diabetes, hyperlipidemia, and some types of cancer (CDC). Exercising or having a good level of physical fitness has many positive effects on health, and preventing or delaying the emergence of chronic diseases [13–16]. As a kind of exercise, the effects of Pilates on body composition parameters have also been examined. In one of them elderly women had decreased body mass index (BMI) as a chronic change [17]. However, one study found that young women did not change their body composition after long-term Pilates training [18].

The effects of Pilates, which is an exercise form that includes whole-body movement, breathing, concentration, precision, and rhythm, on many health parameters have been examined [19]. Rayes, de Lira [20] found significant improvements in body fat, body mass values and oxygen uptake at the ventilatory threshold of over-weight/obese participants after eight weeks of Pilates training. However, the comparative effectiveness of Pilates exercise, slow-controlled breathing exercise and a combination of both have never been examined previously. Therefore, this study aims to compare the effects of Pilates, slow controlled breathing exercises, and a combination of both on HRV, pulmonary function, and body composition in young adult healthy women, and to present information on their effectiveness. Findings of this study will inform the fundamental knowledge and provide new evidence about the selection of appropriate exercise protocols in clinical practice.

## Methods

### Participants

Experimental group participants were selected from those who wanted to register in a private Pilates center in Ankara. The control group was composed among the social circles of the participants included in the study. The mean age was 31.42 ± 6.4 years, body weight 59.4 ± 8.83 kg, body height 163.72 ± 5.98 cm, and BMI 22.18 ± 1.95.

### Study design

**Fig. 1:**
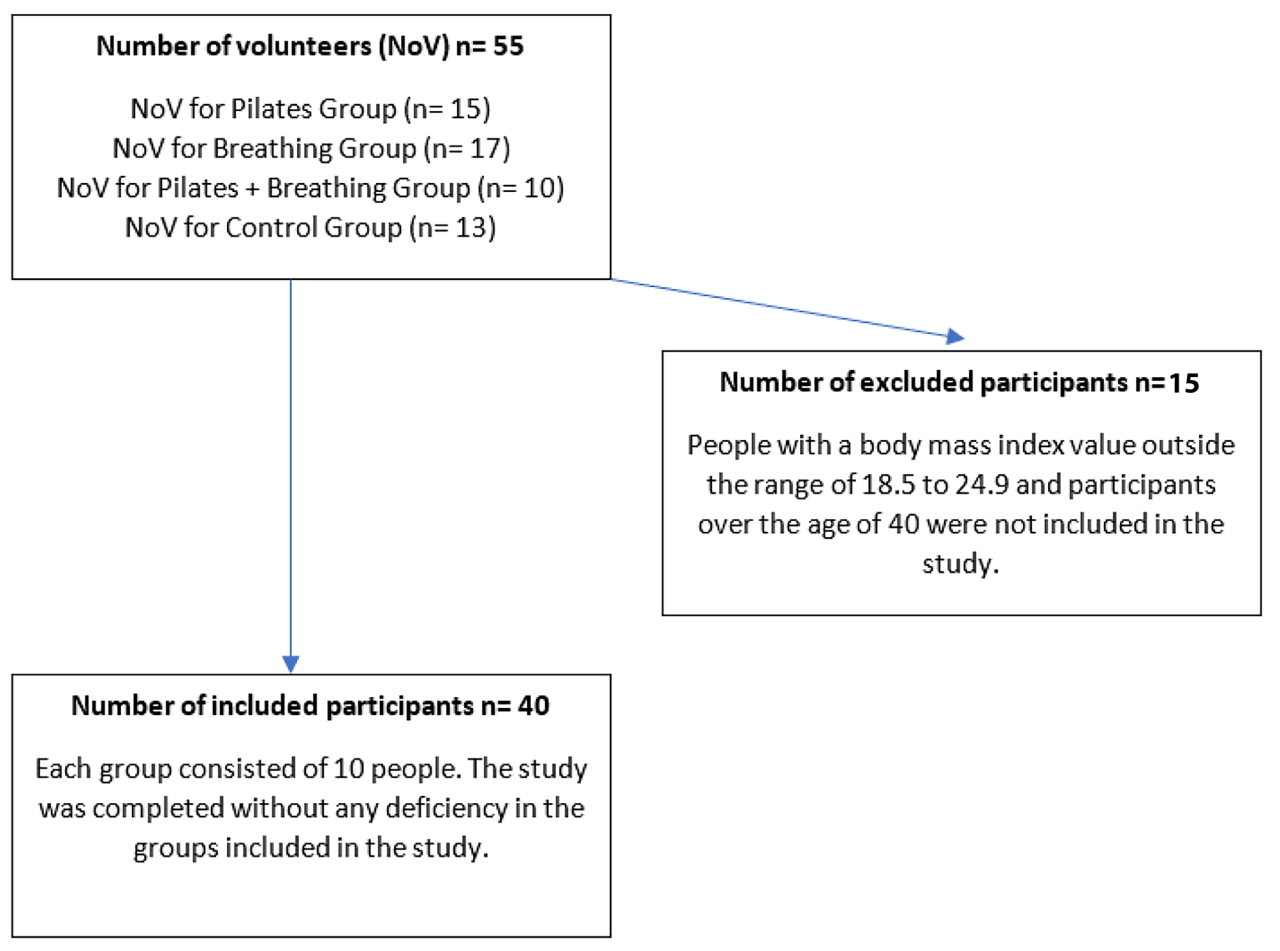
Flow diagram of participants

A total of 40 healthy women voluntarily participated in groups of ten in this study (Fig. 1), in which the effects of 10 weeks of instrumental Pilates exercise on HRV, pulmonary and body composition parameters were examined. G* power software (version 3.0.1) was used to calculate the sample size, with a target effect size 0.30, alpha 0.05 and power 0.95, yielding an estimated sample size of at least 36 participants for the four independent groups [21]. In this quasi-experimental study women whose age is between 18 and 40 and whose BMI is between 18.5 and 24.9 were recruited. Participants who had participated in a regular exercise program for the past six months and had any chronic disease or musculoskeletal injury were excluded from the study. After having the ethical approval from Ankara University Institutional Ethics Committee (09.04.2020/93), and obtaining consent forms, the participants were divided into Pilates group (PG), breathing exercise group (BG), combined Pilates and slow-controlled breathing exercise group (PBG) and control group (CG). All groups participated in pre- and post-test measurements of HRV, pulmonary and body composition. Post-test measurements were taken two days after the experimental protocol. The CG did not participate in any regular physical activity program for 10 weeks, whereas, the PG, PBG and BG groups applied the determined exercise protocol for 10 weeks. Pilates training and breathing exercises were held in a Pilates hall. Before the research, all participants were introduced to the study program and its content. The PG performed Pilates exercise for 50-min a day for two days, and the BG did slow-controlled breathing exercise for 15-min a day for two days. The PBG practiced the breathing exercises after finishing the Pilates session. All exercises were done under supervision of the personal trainer for safety reasons.

### Procedures

Body composition (BC) measurement: PlusAvis 333 (Jawon Medical, SOUTH KOREA) analyse was used to calculate the body composition. Participants were refrained from eating and drinking at least 4 h be-fore the measurement. In addition, they were asked not to take alcohol and diuretic products and not to do any physical activity at least 12 h before the measurement. Before the measurement, the participants were asked to empty their bladders. Besides, all the metal objects were re-moved, and they wore only shorts and sports bra during the measurement. The body weight (BW), percent body fat (PBF), percent body muscle (PBM), and body mass index (BMI) were recorded.

**Fig. 2:**
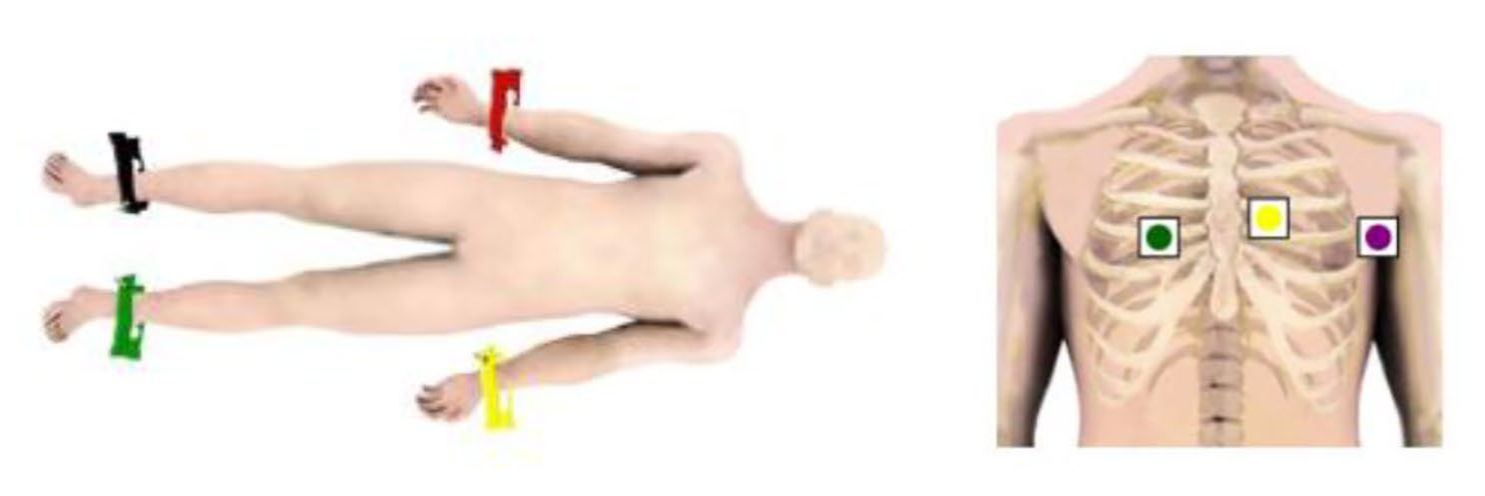
**2a, 2b.** OmegaWave limb and wilson electrodes

Heart rate variability (HRV) measurements: HRV was measured with the OmegaWave 800 device (OmegaWave, Oregon, USA) in a quiet room. All the data were collected between 3 and 5 pm. They were also warned not to exercise, and not to consume alcohol within the 24 h before the test. Participants’ caffeine intake was also limited within the previous 12 h of the test. Measurements were taken in the supine position after the participants had rested for at least 5 min. During the measurements, the participants lay on their backs, wearing only shorts and a sports bra. Then, four Limb and three Wilson electrodes were placed on wrists, ankles and chest appropriately after applying gel (Fig. 2a and b). During the measurement, the participants were warned not to speak or move. The measurement took 5 min. The time- and frequency-domain HRV parameters used in the study were root mean square of successive RR interval differences (RMSSD), the related standard deviation of successive RR interval differences (SDSD), standard deviation of NN intervals (SDNN), total power (TP), high frequency (HF), low frequency (LF), and LF:HF ratio.

**Fig. 3:**
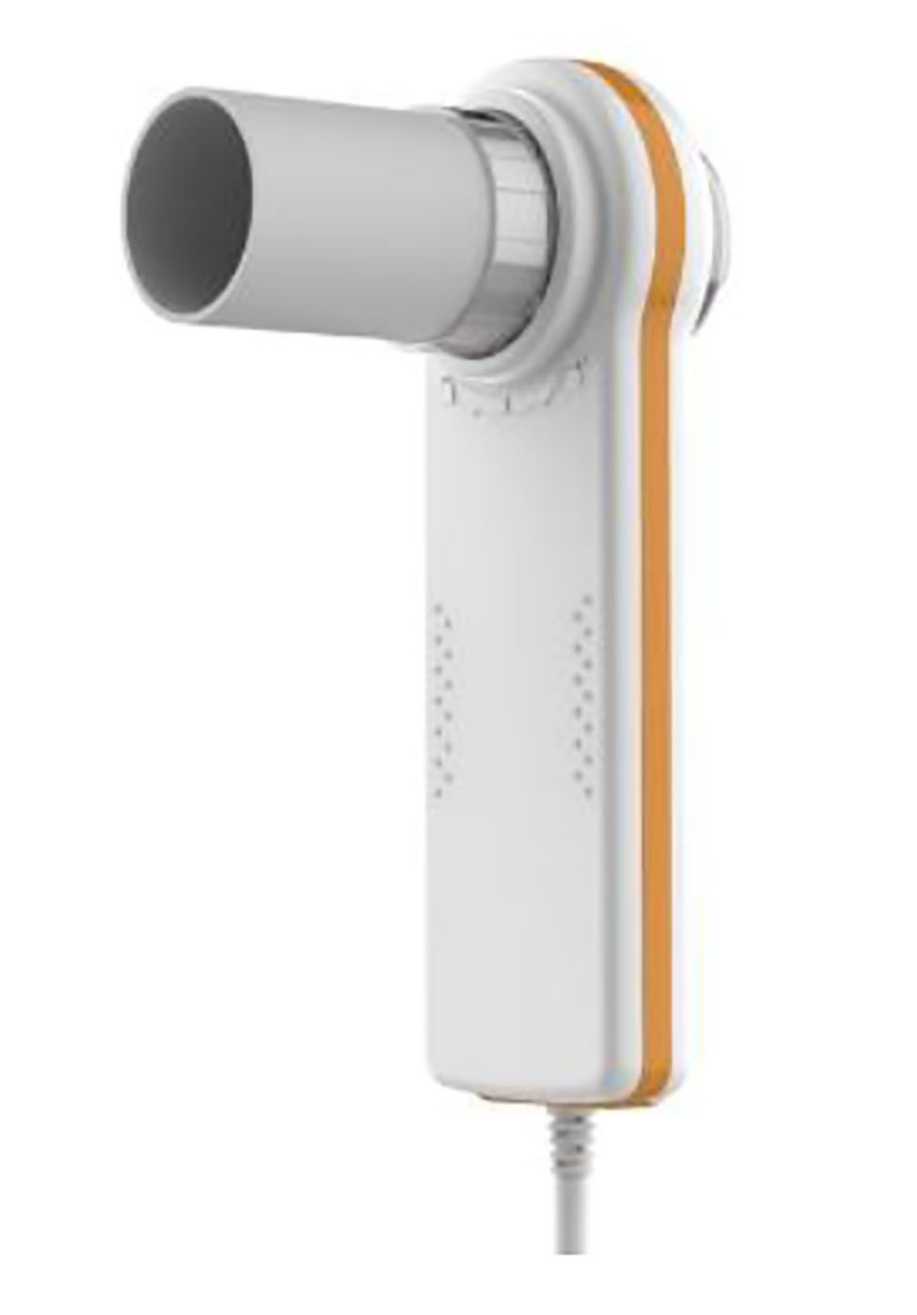
MiniSpir spirometer

Pulmonary function measurements: Lung volumes and capacities were measured with a MIR brand MiniSpir model spirometer (Rome, Italy) (Fig. 3). Forced vital capacity (FVC), forced expiratory volume in 1 s (FEV1), FEV1/FVC (%), peak expiratory flow (PEF), inspiratory capacity (IC), expiratory reserve volume (ERV), vital capacity (VC), tidal volume (TV), maximum voluntary ventilation (MVV), FEV1/VC and minute ventilation (VE) parameters were measured while the participants were in a comfortable sitting position. The whole measurement procedure was performed according to the standards of the American Thoracic Society (ATS)/European Respiratory Society (ERS) [22]. The device was calibrated daily before use. The subjects warned not to eat at least 2 h before the measurement and not wear tight clothing. The disposable mouthpiece of the spirometer was placed in the mouth in the upright position, and the measurement was started after ensuring that the lips were tightly closed. Besides, the nose was closed with an apparatus and air entry and exit from it was prevented. Apparatuses other than the mouthpiece were disinfected before each use. A total of two measurements were taken from each participant with a 5 min rest, and the best measurement was recorded.

Pilates exercises: Each person did the exercise for 50 min, 2 days per week. Reformer, Cadillac, Ladder Barrel, Chair Barrel, Spine Corrector equipment were used in this study. The level of exercise program advanced during the research period. In the first 2 weeks of the study, the Reformer equipment was used since it was easier to control. Participants started using Cadillac equipment in the third week and performed the same movements. Ladder Barrel is used for stretching, usually at the end of the session. In the fifth week, the Chair Barrel, an advanced level equipment considering the balance, was used. Thus, the aim of the 50-minute exercise program was to work on each part of the body to support the physical wellness.

Breathing exercises: The slow and controlled breathing exercises were performed in quiet room by the BG and PBG. Breathing exercises were performed with 10 s of diaphragmatic breathing cycles (6 breaths/min), including 5 s of inhalation and 5 s of exhalation, for 15 min per day, two days a week for ten weeks. All the participants were in supine position one meter apart from each other during each breathing session and were asked not to move or speak during the exercise. To provide the subjects to easily track the 5 s of inhalation and 5 s of exhalation cycles, a large computer screen was projected on the ceiling showing the 5-sec time flow.

### Statistical analyses

All analyses were performed using the SPSS v.22 (SPSS Inc., Chicago, IL, USA). After calculating the descriptive statistics, the distribution and homogeneity of the data were tested. Since the number of participants was below 50 in all four groups, the normality distribution was evaluated with Shapiro-Wilk test. Pre- and post-test mean differences in the data in which the distribution was normal in the in-group statistics were made with the Paired Sample t-Test. A non-parametric Wilcoxon test was used for the data that did not show normal distribution. In the comparison of the mean differences between the groups, One-way ANOVA or Kruskal-Wallis H tests were used, depending on the distribution. The alpha value was accepted as 0.05 in all statistical analyses.

## Results

Body composition parameters and changes in these parameters depending on the application are shown in Table 1.

**Table 1:** Characteristic of body composition across the group

		PG	PBG	BG	CG	*P value*
**BW (kg)**	Pre-test	61.22 ± 4.15	57.14 ± 7.05	61.30 ± 7.32	58.00 ± 3.38	0.06
	Post-test	59.84 ± 3.21	56.40 ± 7.16	63.36 ± 8.55	58.94 ± 4.58	0.11
	***P-value***	0.01*	0.02*	0.01*	0.09	
	%	-2.25	-1.30	3.36	1,62	
**BMI (kg/m2)**	Pre-test	23.09 ± 1.87	21.32 ± 2.26	22.94 ± 1.68	21.38 ± 1.45	0.06
	Post-test	22.32 ± 1.78	21.02 ± 2.17	23.62 ± 2.33	21.71 ± 1.84	0.06
	***P-value***	0.01*	0.01*	0.03*	0.12	
	%	-3,34	-1.41	2.96	1.54	
**PBF (%)**	Pre-test	31.81 ± 4.74	31.37 ± 5.38	34.03 ± 4.33	29.07 ± 4.91	0.17
	Post-test	30.50 ± 3.91	29.95 ± 5.56	34.92 ± 5.06	29.97 ± 5.91	0.10
	***P-value***	0.06	0.00**	0.12	0.19	
	%	-4.12	-4.53	2.62	3.10	
**PBM (%)**	Pre-test	29.14 ± 1.99	28.17 ± 2.54	27.32 ± 1.98	29.69 ± 2.64	0.12
	Post-test	29.72 ± 1.66	28.81 ± 2.26	27.08 ± 2.13	29.16 ± 2.98	0.07
	***P-value***	0.11	0.03*	0.40	0.15	
	%	1.99	2.27	-0.89	-1.79	

BW: Body weight, BMI: Body mass index, PBF: Percent body fat, PBM: Percent body muscle, PG: Pilates group, PBG: Pilates + Slow-Controlled Breathing Exercise Group, BG: Slow-Controlled Breathing Exercise Group, CG: Control group, ES: Effect size, *p < 0.05, **p < 0.01

Table 1 showed that, the BW and BMI were reduced in post-test in both Pilates and Pilates + Slow-Controlled Breathing exercise groups (p < 0.05). The PBF was significantly lower (p < 0.01), and the PBM was higher (p < 0.05) in only Pilates + Slow-Controlled Breathing exercise group. No significant difference between groups was found in the pre-and post-test comparisons.

Tables 2 and 3 display the time- and frequency-domain parameters in HRV, respectively. In accordance with the results, the SDSD, SDNN, TP, HF, and LF were developed in both PBG and BG. However, the percentage increases in PBG were greater, and the RMSSD was recorded higher in only PBG. No change was observed in LF:HF ratio. The PG showed increases in TP and LF. When the intergroup comparisons were examined, a significant difference was observed between PBG and CG in SDNN post-test data (0.014). Similarly, the post-test SDSD, HF, and LF values were higher in PBG than BG (0.010; 0.009; and 0.005 respectively) and CG (0.002; 0.008; 0.005 respectively).

**Table 2:** The comparison of time-domain parameters of HRV.

		PG	PBG	BG	CG	*P-value*
**RMSSD**	Pre-test	47.50 ± 10.88	40.40 ± 13.41	40.70 ± 25.25	41.70 ± 15.80	0.25
	Post-test	62.90 ± 49.02	61.80 ± 37.12	44.40 ± 24.11	39.50 ± 14.14	0.36
	***P-value***	0.63	0.04*	0.39	0.53	
	%	32.42	52.97	9.09	-5.27	
**SDSD**	Pre-test	50.10 ± 9.42	61.30 ± 24.02	43.80 ± 21.03	53.40 ± 19.79	0.23
	Post-test	74.60 ± 55.33	93.10 ± 40.11	59.20 ± 26.40	47.30 ± 20.01	0.00**
	***P-value***	0.05	0.00**	0.00**	0.12	
	%	48.9	51.88	35.16	-11.42	
**SDNN**	Pre-test	48.60 ± 13.70	50.60 ± 20.62	37.20 ± 12.18	44.90 ± 14.06	0.24
	Post-test	63.50 ± 33.99	77.40 ± 27.61	54.30 ± 16.90	41.10 ± 15.81	0.01*
	***P-value***	0.06	0.00*	0.00**	0.24	
	%	30.66	52.96	45.97	-7.80	

RMSSD: Root mean square of successive RR interval differences, SDSD: The related standard deviation of successive RR interval differences, SDNN: Standard deviation of NN intervals

*p < 0.05, **p < 0.01

**Table 3:** The comparison of frequency-domain parameters of HRV.

		PG	PBG	BG	CG	*P-value*
**TP**	Pre-test	852.40 ± 589.27	946.1 ± 585.28	787.1 ± 1201.91	697.2 ± 563.48	0.23
	Post-test	1585.60 ± 1896.90	2203.70 ± 1502.74	983.40 ± 1296.57	623.10 ± 355.58	0.01*
	***P-value***	0.04*	0.00**	0.00**	0.95	
	%	86.02	132.85	24.94	-10.63	
**HF**	Pre-test	472.80 ± 454.98	603.6 ± 451.30	500.90 ± 889.38	393.8 ± 414.28	0.50
	Post-test	1022.80 ± 1562.13	1555.30 ± 1391.12	613.20 ± 975.49	370.50 ± 254.45	0.05*
	***P-value***	0.05	0.00**	0.00**	0.79	
	%	116.33	157.67	22.42	-5.92	
**LF**	Pre-test	276.60 ± 151.99	252.3 ± 136.53	223.0 ± 305.81	214.8 ± 167.83	0.25
	Post-test	427.00 ± 330.45	461.00 ± 116.40	290.10 ± 326.82	179.30 ± 123.51	0.00**
	***P-value***	0.03*	0.00**	0.00**	0.15	
	%	54.37	82.72	30.09	-16.53	
**LF:HF ratio**	Pre-test	0.95 ± 0.79	0.72 ± 0.58	0.86 ± 0.57	0.65 ± 0.16	0.63
	Post-test	0.87 ± 0.56	0.58 ± 0.50	0.83 ± 0.55	0.59 ± 0.16	0.38
	***P-value***	0.64	0.20	0.79	0.25	
	%	-8.42	-19.44	-3.49	-9.23	

TP: Total power, HF: High frequency, LF: Low frequency, LF:HF: LF:HF ratio

*p < 0.05, **p < 0.01

Another aim of the study was to observe the changes in respiratory parameters. Table 4 showed that, the most obvious changes were noted in the PBG group. FVC, FEV1, VC, IC, TV, MVV, and VE were improved after the 10-week implementation in PBG. Similarly, PG developed VC and TV. Only significant enhancements in BG were found in PEF and ERV.

**Table 4:** The pre- and post-test results and changes of respiratory parameters

		PG	PBG	BG	CG	*P-value*
**FVC**	Pre-test	3.81 ± 0.13	4.0 ± 0.14	4.0 ± 0.39	3.8 ± 0.14	0.84
	Post-test	4.16 ± 0.28	4.4 ± 0.10	4.3 ± 0.10	3.8 ± 0.18	0.18
	***P-value***	0.18	0.01*	0.26	0.96	
	%	9.19	10.0	7.50	0	
**FEV**_**1**_	Pre-test	3.21 ± 0.93	3.4 ± 0.78	3.1 ± 0.23	3.3 ± 0.12	0.56
	Post-test	3.28 ± 0.10	3.6 ± 0.06	3.2 ± 0.21	3.2 ± 0.13	0.11
	***P-value***	0.42	0.01**	0.76	0.02*	
	%	2.18	5.88	3.23	-3.03	
**FEV**_**1**_ **/FVC**	Pre-test	84.5 ± 2.09	84.9 ± 2.55	81.7 ± 3.6	87.5 ± 1.7	0.48
	Post-test	81.3 ± 4.3	82.4 ± 1.85	73.5 ± 3.0	85.0 ± 3.4	0.10
	***P-value***	0.44	0.47	0.24	0.40	
	%	-3.79	-2.94	-10.04	-2.86	
**VC**	Pre-test	4.0 ± 0.18	4.1 ± 0.29	3.9 ± 0.30	3.8 ± 0.18	0.84
	Post-test	4.2 ± 0.13	4.7 ± 0.12	4.0 ± 0.26	3.8 ± 0.19	0.01*
	***P-value***	0.02*	0.03*	0.30	0.92	
	%	5.0	14.63	2.56	0	
**FEV**_**1**_ **/VC**	Pre-test	81.4 ± 10.47	85.8 ± 17.13	81.7 ± 16.68	88.9 ± 7.70	0.38
	Post-test	77.8 ± 7.24	77.09 ± 4.14	80.02 ± 11.34	86.2 ± 8.06	0.11
	***P-value***	0.16	0.13	0.50	0.79	
	%	-4.42	-10.05	-2.06	-3.04	
**PEF**	Pre-test	6.8 ± 0.60	6.18 ± 0.56	5.4 ± 0.31	7.1 ± 0.55	0.13
	Post-test	7.3 ± 0.36	7.42 ± 0.30	5.9 ± 0.28	6.5 ± 0.41	0.01*
	***P-value***	0.26	0.78	0.01*	0.02*	
	%	7.35	20.6	9.26	-8.45	
**IC**	Pre-test	2.4 ± 0.23	2.4 ± 0.32	2.5 ± 0.16	2.1 ± 0.20	0.22
	Post-test	2.6 ± 0.21	3.1 ± 0.12	2.6 ± 0.12	2.3 ± 0.14	0.02*
	***P-value***	0.20	0.00**	0.07	0.95	
	%	8.33	29.17	4.31	9.52	
**ERV**	Pre-test	1.45 ± 0.12	1.65 ± 0.38	1.30 ± 0.14	1.61 ± 0.17	0.62
	Post-test	1.70 ± 0.08	1.93 ± 0.04	1.51 ± 0.10	1.59 ± 0.17	0.10
	***P-value***	0.08	0.13	0.01*	0.13	
	%	17.24	16.97	16.15	-1.24	
**TV**	Pre-test	0.81 ± 0.11	0.93 ± 0.12	0.74 ± 0.06	0.64 ± 0.46	0.18
	Post-test	0.96 ± 0.92	1.20 ± 0.06	0.80 ± 0.07	0.64 ± 0.49	0.00**
	***P-value***	0.00**	0.00**	0.24	0.97	
	%	18.52	29.03	8.11	0	
**MVV**	Pre-test	93.9 ± 7.42	95.8 ± 5.9	75.9 ± 5.2	87.3 ± 7.7	0.11
	Post-test	97.2 ± 8.25	111.3 ± 5.8	75.2 ± 5.5	77.4 ± 6.2	0.00**
	***P-value***	0.50	0.03*	0.01*	0.01*	
	%	3.51	16.18	-0.92	-11.34	
**VE**	Pre-test	15.4 ± 1.68	14.5 ± 1.2	13.5 ± 1.9	12.4 ± 1.29	0.58
	Post-test	16.5 ± 0.99	21.0 ± 0.4	15.8 ± 1.6	13.8 ± 1.56	0.00**
	***P-value***	0.51	0.00**	0.12	0.20	
	%	7.14	44.83	17.4	11.29	

FVC: Forced * p < 0.05, ** p < 0.01 vital capacity, FEV_1_: Forced expiratory volume in 1 s, VC: Vital capacity, PEF: Peak expiratory flow, IC: Inspiration capacity, ERV: Expiratory reserve volume, TV: Tidal volume, MVV: Maximum voluntary ventilation, VE: Minute ventilation

* p < 0.05, ** p < 0.01

FVC: Forced vital capacity, FEV_1_: Forced expiratory volume in 1 s, VC: Vital capacity, PEF: Peak expiratory flow, IC: Inspiration capacity, ERV: Expiratory reserve volume, TV: Tidal volume, MVV: Maximum voluntary ventilation, VE: Minute ventilation.

## Discussion

This study aimed to compare the effectiveness of 10-week slow-controlled breathing exercise, equipment-based Pilates, and when both were performed together on HRV, pulmonary function, and body composition in healthy women. Several Pilates and its influence on body composition have been well studied. Fourie, Gildenhuys [23] examined the effects of 8-week mat Pilates in older women when it is done 3 times a week and one hour per session. At the end of the intervention process they found significant decrease in body fat and increase in lean body mass. In another study comparing the effects of mat Pilates with low-load-high-repetitions resistance training, participants performed an exercise program three days a week and one hour a day for 3 months. Results showed that mat Pilates focusing on breathing, concentration, control and precision was ineffective on any BC parameters [24].

Aibar-Almazán, Martínez-Amat [17] performed a 12-week Pilates exercise, which started with sitting and standing positions and later added floor exercises, on elderly women for one hour a day, two days a week. Only significant change in BC parameters was the decrease in BMI. The skeletal muscle mass did not alter. Another study compared the pre- and post-intervention results of 15-week Pilates program with two 50-minute session per week. As a result, young, healthy sedentary women did not show any improvements in BC parameters [18]. Lastly, Tolnai, Szabó [25] demonstrated that 15-week Pilates program was effective on providing an increase in muscle mass even if it is done once a week for hour per session in young sedentary women. In the current study, there was no change in BC parameters in the breathing exercise group besides the control group. However, BMI and body weight decreased in PG and PBG, and percent body fat decreased in PBG. Current research results are similar to some studies in the literature. It can be said that the difference between the studies examined in the literature is due to the research group, the duration and frequency of Pilates, and more importantly, the difference in the Pilates content applied.

Comparing changes in HRV and respiratory parameters concerning physical and breathing exercises was the main point of the present study. Significant changes in both time- and frequency-domain parameters of HRV were observed. The PG group showed improvement only in TP and LF (p < 0.05). The SDSD, SDNN, TP, HF, and LF were improved in PBG and BG (p < 0.01). And at last, PBG was observed as the only group in which the RMSSD increased (p < 0.05). These results can be evaluated in two ways. The first important point is that these changes affect the ANS by im-proving the parasympathetic nervous system. The increase in time-domain parameters such as SDSD, SDNN, and RMSSD is correlated with increased parasympathetic activity [2]. Changes in the frequency-domain parameters of HRV, such as TP, HF, and LF, also indicate an increase in parasympathetic activity. The total power is indicative of the sum value of HF, LF and VLF in short-term recordings Shaffer, McCraty [2] as used in this research. LF, on the other hand, can be produced by both the sympathetic and parasympathetic systems [8] or primarily by the parasympathetic system [26]. The HF is highly correlated to parasympathetic activity [27], and high HF values reflect a good level of cardio-vascular health [28]. In accordance with the findings, it could be concluded that positive effects on ANS after both physical and breathing exercises were observed.

The second important point is that slow-controlled breathing exercises alone are more effective on HRV than Pilates alone, but its effect is increased when combined with Pilates exercise. Sürücü, Güner [29] investigated the effects of six-week slow and controlled breathing exercises on HRV, and found significant improvement in LF:HF ratio and LFnu parameters in healthy male and females. Their recommendation was that the training period should be at least eight weeks to see clearer alterations in HRV. The present paper studied for ten-week and more changes were observed in HRV. In both studies, the breathing cycle was performed with a total of 10-second cycles consisting of 5-second inhale and 5-second ex-hale phases for 15 min per day. Similarly [11] reported increases in parasympathetic activity after 3 months of slow and con-trolled breathing exercises when compared with a fast-breathing group. In this paper, the breathing exercises were done for 18 s per cycle, and performed for about 30 min per day in healthy adults. In the current study, PBG did have the most significant improvements in vagal tone with an increase in RMSSD in addition to the increases in SDSD, SDNN, TP, HF, and LF observed in both BG and PBG. There are also studies in the literature examining the effects of physical exercises on HRV and therefore on the ANS. In a study comparing the group whose individual training intensity was determined over HRV and the groups that were given standard loads, it was reported that at the end of eleven weeks, HR recovery time was shortened in all groups, and RMSSD in-creased only in the group whose training intensity was determined over HRV in healthy sedentary women [30]. Kim, Kim [31] compared the effectiveness of 8-week of moderate- (60% of heart rate re-serve) and high-intensity (75% of heart rate reserve) training on HRV variables in healthy males. They declared that the high-intensity group showed significant increases in RMSSD, pNN50, HF, SD1 and SD1:SD2, and decreases in LF:HF ratio and LF. Cavina, Silva [3] found significant alterations in LF, SDNN, and SD1 parameters after 12-week of Pilates training in healthy men. In this study, the difficulty of the Pilates exercises was increased depending on time as in the current research, and similar results were found in HRV parameters. However, using equipment-based Pilates in the current research may be the reason for the change in parameters such as SDSD and TP in addition to LF and SDNN. In another study, pNN50 was found to be high after three days a week and a total of 12 weeks of mat Pilates exercise in postmenopausal women [32].

Similar to the changes observed in HRV, pulmonary function parameters also show that Pilates and breathing exercises are more effective when performed together. The covariation of respiratory and HRV parameters supports this. HF, as an indicator of vagal tone is usually called the respiratory band since its variations related to the respiratory cycle. This condition, known as respiratory sinus arrhythmia (RSA), is explained by increased heart rate during inspiration and decreased during expiration. It is suggested that the magnitude of the oscillation is variable but will increase with slow breathing exercises [2]. For this reason, it can be stated that slow breathing exercises cause an increase in HF and other related parameters, as in the current research. SDNN is affected by parasympathetically mediated RSA when it is recorded during resting particularly when slow breathing is performed. Although the relationship between RMSSD and RSA is not clearly stated, RMSSD is closely related to HF and is also the main parameter of HRV that indicates vagal tone [2]. However, even though the LF can be affected by different parameters, it more reflects the vagal effect, especially when the respiratory rate drops below 8.5 bpm or 7 s [27]. In the present study the PBG was the only group in which the FVC, VC, IC (p < 0.05), FEV1, TV, MVV, and VE (p < 0.01) were im-proved. The PG showed enhancements in VC (p < 0.05) and TV (p < 0.01), and the BG only in ERV (p < 0.05). Lazovic-Popovic, Zlatkovic-Svenda [5] investigated some lung volume and capacities among swimmers, football players and control group, and swimmers were found to have the highest in VC, FVC, FEV1, FEV1/FVC, PEF, and MVC values. In addition, the values of the football players were better than the control group. They declared that regular training develops the lung functions. The results of the current research both support this and provide additional information. According to the present findings even the 10-week of equipment-based Pilates exercises had some positive effects on respiratory parameters, but its effectiveness is clearly increased when breathing exercises added. Only one study in the literature has a slightly similar research method to the cur-rent study. Alvarenga, Charkovski [6] examined the inspiratory muscle training combined with Pilates exercise on pulmonary functions in elderly women. They had Pilates, Pilates + inspiratory training and control groups, used Cadillac, Combo Chair and Reformer equipment for Pilates exercise, and performed only inspiratory training consisting of 30 inspiratory efforts in two sets in each session. Although breathing exercises were different, the research was done in the elderly, and no group only did breathing exercises, the results are worth comparing with the current research. As a result, they concluded that Pilates exercise associated with inspiratory muscle training has more significant effects on lung functions. Han and Kim [7] compared the influence of breathing exercise with its combination with upper body exercises in healthy young adults, and found increment in FVC in both groups and in FEV only in the combined exercise group. It is understood that these results are in line with the current research.

The strength of this study is that it is the first in which the effects of equipment-based Pilates, slow-controlled breathing exercises, and a combination of both were examined in healthy sedentary women. The findings proved that, besides their positive influences on body composition, equipment-based Pilates exercises were more efficient in developing HRV and respiration parameters when done with slow-controlled breathing exercises. Therefore, doing breathing exercises as a non-pharmacological method together with physical activity at least twice a week can be preferred because of their positive effects on health.

### Limitations

There were some limitations in this study. First, the number of participants could have been higher. Relatedly, the second limitation was that the sample distribution revealed that the study only recruited women, which may affect the generalizability of the finding. Another limitation was that the heart rate variability was evaluated by short-term measurement in the current research. We could have obtained better results if we had the opportunity to measure it over a 24-hour period by using an electrocardiography Holter. Finally, although the research was carried out with instrumental Pilates, the equipment used was specified and the load was gradually increased, the inability to use the same training program creates a difficulty in comparison with other studies as a general limitation of such studies.

## Conclusions, future directions and practical implications

The main findings in this study on highlights the ample effect of combined slow-controlled breathing and Pilates exercise on HRV, pulmonary function and body composition which has important implications for health promotion. Since breathing exercises in addition to Pilates exercise can lead to significant improvements in body composition, HRV, and pulmonary function, it should be considered as an alternative physical fitness program for female individuals. Especially in the society, women can be encouraged to participate in sports and Pilates, and health can be improved. Ministries of health and/or local governments can develop policies that will ensure the spread of equipment-based Pilates exercises to the community. It is known that a physically sedentary life is associated with many cardiovascular, metabolic and mental disorders and that exercise has positive effects on health not only in the healthy population but also in people with chronic diseases [33, 34]. For instance, [35] reported that both high-intensity interval training or moderate-intensity continuous training have similar beneficial effects after coronary artery bypass graft. Considering that one out of every three people worldwide has a chronic illness [36], it can be thought that participation in such activities will alleviate the burden on the health system. Expanding the research group in future studies and conducting similar studies with larger sample numbers in men and/or elderly groups may increase the generalizability of the results. Future studies may also record HRV for 24 h via electrocardiography Holter, which may provide a clearer understanding of the effects of such exercises on the functional functioning of the heart.

## Data Availability

The data presented in this study are available on request from the corresponding author. The data are not publicly available due to restrictions on privacy.
